# Lean Body Mass in the Prediction of Bone Mineral Density in Postmenopausal Women

**DOI:** 10.1089/biores.2018.0025

**Published:** 2018-10-10

**Authors:** Bolaji Lilian Ilesanmi-Oyelere, Jane Coad, Nicole Roy, Marlena Cathorina Kruger

**Affiliations:** ^1^ School of Food and Nutrition, Massey University, Palmerston North, New Zealand.; ^2^ Riddet Institute, Massey University, Palmerston North, New Zealand.; ^3^ Food Nutrition and Health Team, Food and Bio-Based Products Group, AgResearch Grasslands, Palmerston North, New Zealand.; ^4^ High-Value Nutrition National Science Challenge, Auckland, New Zealand.

**Keywords:** bone mineral density, fat mass, lean mass, postmenopausal women

## Abstract

Owing to conflicting results of the association between body composition and bone mineral density (BMD), we investigated the relationship between fat mass (FM), lean mass (LM), and BMD in New Zealand postmenopausal women. We hypothesized that increased LM will indicate a higher BMD. A cross-sectional study was performed examining the associations between body composition, anthropometric measures, activity energy expenditure, and bone health status (using dual-energy X-ray absorptiometry [DXA]). A total of 127 healthy postmenopausal women aged between 54 and 81 years. Both FM and LM were significantly associated with BMD at all sites. However, LM, not FM, was the strongest predictor of femoral neck (FN) BMD (β = 0.497, *p* < 0.001), hip BMD (β = 0.495, *p* < 0.001), spine BMD (β = 0.449, *p* < 0.001), and whole body BMD (β = 0.406, *p* < 0.001). Age was negatively associated with FN and hip BMD. LM was positively associated with FN, spine, hip, and whole body BMD. Our findings suggest the need to increase LM rather than FM highlighting the importance of physical activity for this age group.

## Introduction

Postmenopausal osteoporosis is a disease of public health concern and due to its debilitating nature affects the aged, especially elderly women. Osteoporosis in postmenopausal women is associated with a reduction in estrogen levels, which consequently results in the acceleration of bone fragility and fracture.^1^ The World Health Organization (WHO) defines osteoporosis as a disease that is characterized by low bone mass and microarchitectural deterioration of bone tissues, leading to bone fragility and increased fracture risk. The diagnosis of osteoporosis according to WHO may be obtained from one or more of the following regions: total hip, femoral neck (FN), and lumbar spine (LS).^2^

The disease state may result in fracture, which could subsequently lead to lack of independence and mobility. Body composition is an important part in the determination of bone mineral density (BMD) and bone mineral content (BMC) as well as osteoporotic status. Body weight tends to have the capacity to elevate bone mineral status due to its ability to exact mechanical force and action on the host. Lean mass (LM), fat mass (FM), and bone mass are the three components of body weight found to be associated with bone status.^3^ LM, FM, bone mass, and water together accounts for ∼90–95% of the body weight.^4^

Many epidemiological studies have reported and suggested that both FM and LM may affect bone mass status especially in the aged group.^5,6^ Adipose tissue is metabolically active; therefore, its effects on the bone or skeleton may be regulated by the weight-bearing effect as well as nonweight-bearing effects.^5^ Examples of the nonweight-bearing effect include the hormonal metabolism of the adipocytes, such as leptin, insulin-like growth factor 1, and several cytokines. It has thus been reported to a degree of conclusion that weight-bearing and resistance-type physical activity has a positive effect and can serve as a measure for the prevention of osteoporosis.^5,7^

Studies have had a controversial report on whether being overweight and obese results in a detrimental or protective effect on bone health. Both fat and bone cells originate from the same bone marrow stem cells,^8,9^ and it is well known that physical inactivity and ageing induces both obesity and osteoporosis.^10^ In addition, these two disorders synergistically induce functional impairments and physical disabilities, which suggest a complex effect of obesity on bone health. The protective effect of obesity on bone mass has, therefore, been termed “obesity paradox” or “reverse epidemiology.”^5,11^

The comparative contribution of the body fat and LM (or fat-free mass) to BMD variation has been controversial based on the original research findings available. Some studies^12–14^ have reported that LM, not FM, is associated with bone mass, whereas others^5,15^ have found that FM, not LM, is important in the determination of BMD. Whereas some have indicated that both FM and LM can equally serve as a predicting factor for BMD.^16^ Furthermore, some studies have reported that LM is of more importance than FM in premenopausal women and FM is more significant than LM in postmenopausal women.^17,18^ However, other studies have shown that LM was associated with BMD in both pre- and postmenopausal women.^8,19^ Furthermore, some studies made an observation that FM was associated with BMD in men <50 years; meanwhile, this was not the case in women and men >50 years.^5^ The inconsistency in the findings may be due relatively to methodology and inadequately powered study design. Altogether, Ho-Pham et al.^6^ suggested that age, ethnic group, and gender play a major role in the relative contribution of body composition parameters to BMD as well as the site of measurement.

Owing to the presence of conflicting findings in the relationship between body composition and bone density, this study will shed light in terms of New Zealand postmenopausal women's perspective. Two research questions guided this study: (1) How are body composition measures such as fat or LM related to regional and whole body measures such as femoral, hip, spine, and whole body BMDs? (2) How does these regional and whole body measures relate to anthropometric variables such as weight and body mass index (BMI) as well as quantitative ultrasound sonometry (QUS) T-score and the activity energy expenditure (AEE)?

## Materials and Methods

### Study design

A total of 127 postmenopausal women aged between 54 and 81 years participated in the “Bugs‘n’Bones” study that took place in the Human Nutrition Research Unit of Massey University, Palmerston North campus from June to December 2017. Sample size was calculated using G*Power software version 3.0.10 and 88 subjects were required for a 90% power and an α of 5% for *t*-test. A total of 150 was required based on osteoporosis incidence ratio of 3:1 in women. In this cross-sectional study, two subjects were excluded from the study, one due to a ketogenic diet and the other due to health conditions. Subjects were recruited by advertisement on campus, the Whanganui Chronicle, and by using a recruitment agency, Trial Facts (https://trialfacts.com/). The inclusion criteria were confirmed as menopause of at least 5 years based on no menstruation. Exclusion criteria were presence of any systemic disease, food intolerances that affect the gastrointestinal tract, smokers, and high intake of alcohol. Subjects with significant weight loss or weight gain within the past year were excluded. All participants completed the New Zealand Physical Activity Questionnaire (NZPAQ)^20^ and the AEE was calculated. All subjects were free living and apparently healthy. Written informed consent was obtained from subjects before commencing data collection. The study was registered with the Australian New Zealand Clinical Trials Registry with the number ACTRN12617000802303. This study was also approved by Massey University Human Ethics Committee: Southern A, Application 17/17, following the Helsinki Declaration of 1975, as revised in 2008.

### Anthropometric and body composition measurements of the subjects

Body weight of subjects was measured using the Detecto 437 eye-level weigh beam physician scale to the nearest 0.2 kg and standing height was measured using a stadiometer to the nearest 0.1 cm wearing light clothes and no shoes on. The BMI was calculated as weight divided by height squared (kg/m^2^). Waist to hip ratio was determined by measuring the waist circumference (WC) and hip circumference (HC) to the nearest 0.1 cm using a nonstretchable tape. Waist to hip ratio was calculated as a marker of abdominal obesity. Stiffness index, QUS T-score, and Z-score of the nondominant heel scan were measured using the GE Lunar Achilles II Portable Bone Densitometer.

Body composition measurements, FM, LM, and fat percentage were measured and analyzed using the Hologic QDR series Discovery A Bone densitometry (dual-energy X-ray absorptiometry [DXA]). BMD was measured at the FN, LS (L1–L4), trochanter, Ward's triangle, and total hip. The DXA machine was calibrated every morning for all the measurements and at the end of each day. The *in vivo* reproducibility of the coefficient of variation ranged between 0.34% and 0.70% for all measured sites. The reported BMD values were calculated as means of four measured values from L1 to L4. Apex System Software version 4.5.3 was used for analyzing the DXA scans. Osteoporosis was defined as a T-score ≤2 · 5 and osteopenia as T-score between −1.0 and −2.5 according to the WHO criteria.^2^

### Statistical analyses

IBM SPSS version 25 (IBM Company, Armonk, NY) was used for all statistical analyses. The outcome variables used were BMD of whole body and at skeletal sites. The values of all variables for the whole body and regional sites were presented as mean (M) ± standard deviation (SD). Comparisons of the mean values of two groups of healthy and osteopenic/osteoporotic subjects classified according to their spine T-scores were analyzed by independent *t*-test as parametric variables. The mean difference of other groups of subjects with BMI <25 kg/m^2^ and BMI ≥25 kg/m^2^ were compared using independent *t*-test. Correlation analyses of the whole body, regional sites BMD, and T-scores with the independent variables such as age, weight, BMI, AEE, and QUS T-score were performed to obtain the Pearson's correlations. Stepwise multiple linear regression analysis was used to obtain the determinants/predictors for the outcome variables. All *p*-values were reported significant at 0.05 or less.

## Results

Table 1 shows the demography, body composition, and lifestyle characteristics of the 125 women studied. The BMI of the women ranged from 14.9 to 44.0 kg/m^2^. According to the WHO classification, 2.4% of the women were underweight, 34.4% were of normal weight, 48% were overweight, and 15.2% were obese.

**Table 1.** **Subjects' Baseline Characteristics and Anthropometric Variables**

	Mean ± SD	Range	
Parameters	*n* = 125	Min	Max
Age (years)	62.6 ± 4.5	54.0	81.0
Weight (kg)	69.3 ± 11.2	43.0	110.8
Height (cm)	162.3 ± 5.3	149.1	175.4
BMI (kg/m^2^)	26.3 ± 4.2	14.9	44.0
WC (cm)	80.8 ± 10.8	57.0	110.0
HC (cm)	99.3 ± 7.6	78.0	122.5
Spine area (cm^2^)	57.4 ± 6.0	23.7	71.2
Spine BMD (g/cm^2^)	0.94 ± 0.15	0.5	1.3
Spine BMC (g)	54.2 ± 11.4	26.7	82.6
Spine T-score	−0.9 ± 1.4	−4.6	2.6
FN area	5.0 ± 0.4	3.9	6.3
FN BMC (g)	3.6 ± 0.5	2.4	5.2
FN BMD (g/cm^2^)	0.71 ± 0.10	0.5	1.0
Hip area (cm^2^)	40.0 ± 3.4	27.2	44.3
Hip BMC (g)	29.9 ± 5.1	19.0	44.2
Hip BMD (g/cm^2^)	0.85 ± 0.11	0.6	1.2
Hip T-score	−0.7 ± 1.0	−2.5	2.1
Whole body area (cm^2^)	1952.6 ± 143.2	1641.3	2387.6
Whole body BMC (g)	2207.0 ± 333.7	1618.3	3385.5
Whole body BMD (g/cm^2^)	1.13 ± 0.11	0.9	1.5
Whole body TFM (kg)	29.4 ± 8.3	6.4	56.5
Whole body TLM (kg)	40.6 ± 4.5	30.7	57.3
Whole body total mass (kg)	70.0 ± 11.2	43.1	112.6
Whole body total %fat	41.2 ± 6.5	14.8	52.8
Stiffness index	88.9 ± 13.6	54.0	137.0
QUS T-score	−0.7 ± 0.9	−2.9	2.3
QUS Z-score	0.8 ± 0.8	−1.2	4.0
AEE (cal/min)	3056.10 ± 10,793.96	0.0	101,207.2

AEE, activity energy expenditure; BMC, bone mineral content; BMD, bone mineral density; BMI, body mass index; FN, femoral neck; HC, hip circumference; Max, maximum; Min, minimum; QUS, quantitative ultrasound sonometry; SD, standard deviation; TFM, total fat mass; TLM, total lean mass; WC, waist circumference.

**FIG. 1.**
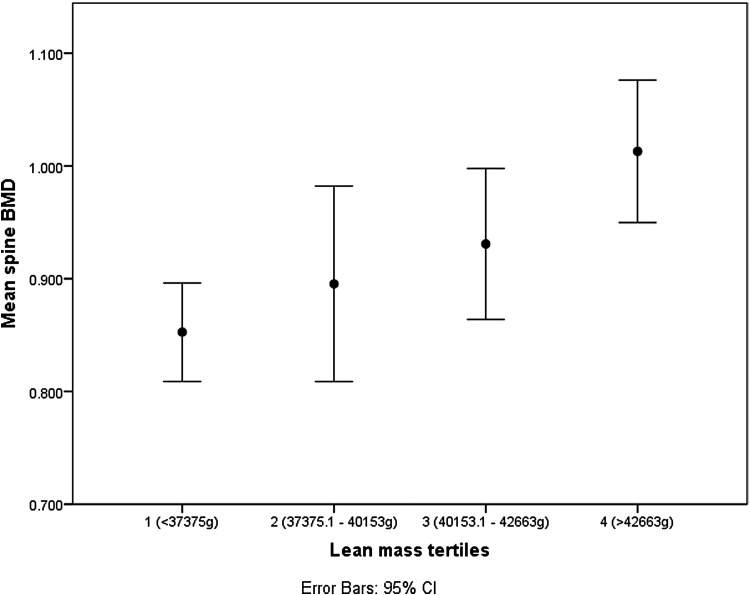
Relationship between spine BMD and LM tertiles. BMD, bone mineral density; CI, confidence interval; LM, lean mass.

**FIG. 2.**
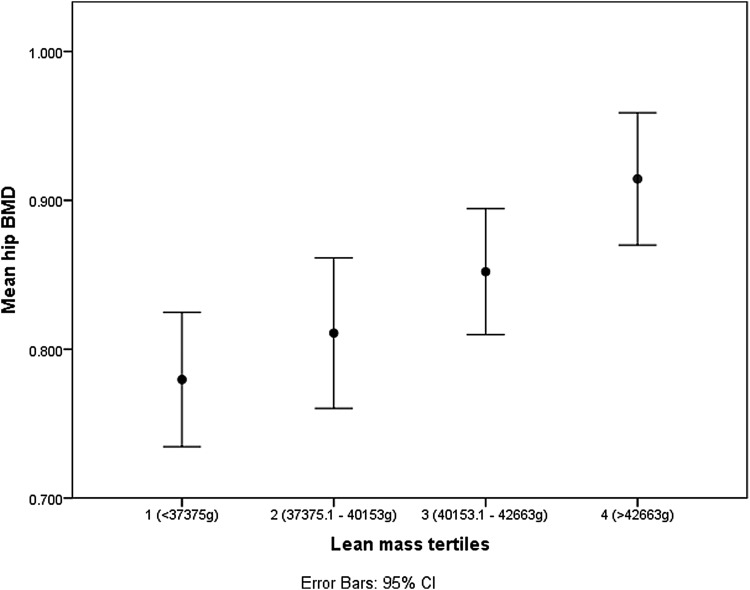
Relationship between hip BMD and LM tertiles.

**FIG. 3.**
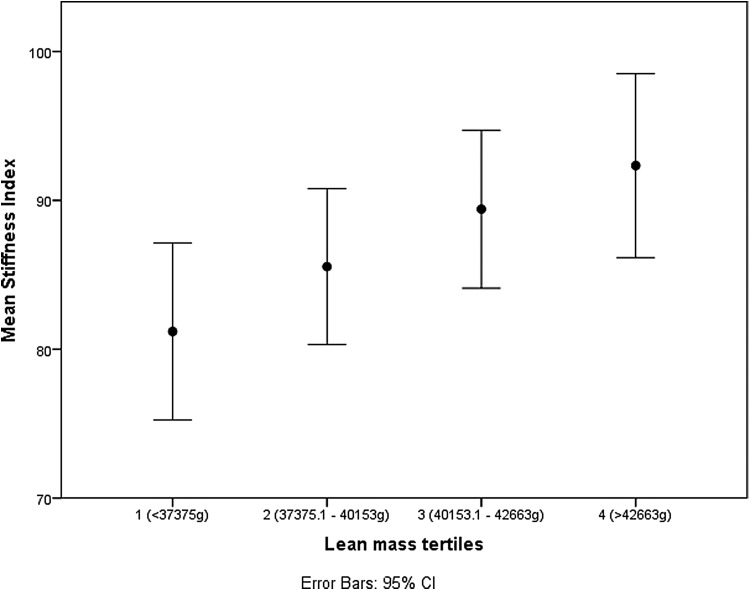
Relationship between stiffness index and LM tertiles.

Figures 1–3 show the spine BMD, hip BMD, and stiffness index with respect to the quartile distributions of LM. The BMD and stiffness index increased linearly with an increase in LM in all the three instances.

In Table 2, to test the hypothesis that osteopenic/osteoporotic and women with normal bone mass have equal mean body compositions, an independent *t*-test was performed. In this selected population, based on the T-score, there were 60 women with normal bone mass (T-score ≥ −1.0) and 65 women with osteopenia/osteoporosis (T-score < −1.0). The osteopenic/osteoporotic group of women were slightly older, shorter, and thinner. They had lower BMC, BMD, bone area, body mass, body mass components, and AEE than the healthy women (Table 2). Osteopenic/osteoporotic women (M = 62.9, SD = 4.0) and those with normal bone mass (M = 62.3, SD = 5.0) did not differ significantly according to their age (*t*[123] = −0.74, *p* = 0.463). Conversely, concerning FM and LM, osteopenic/osteoporotic women (M = 26.7, SD = 7.6 and M = 38.8, SD = 3.6) and women with normal bone mass (M = 32.2, SD = 8.2 and M = 42.6, SD = 4.6) were significantly different; *t*(123) = 3.87, *p* < 0.001 and *t*(123) = 5.11, *p* < 0.001, respectively.

**Table 2.** **Comparison of Subjects' Anthropometric and Dual-Energy X-Ray Absorptiometry Data Without and With Osteoporosis**

	Women without osteoporosis (*n* = 60)	Women with osteopenia/osteoporosis (*n* = 65)	Independent *t*-test
Parameters	Mean ± SD		*p*
Age (years)	62.3 ± 5.0	62.9 ± 4.0	0.463
Weight (kg)	74.0 ± 10.9	64.9 ± 9.8	<0.001
Height (cm)	163.2 ± 5.9	161.5 ± 4.6	0.084
BMI (kg/m^2^)	27.9 ± 4.3	24.9 ± 3.6	<0.001
WC (cm)	84.7 ± 10.9	77.2 ± 9.5	<0.001
HC (cm)	101.7 ± 6.8	97.2 ± 7.8	0.001
Spine area (cm^2^)	58.9 ± 6.8	56.1 ± 4.9	0.009
Spine BMD (g/cm^2^)	1.07 ± 0.10	0.82 ± 0.08	<0.001
Spine BMC (g)	63.0 ± 9.3	46.1 ± 6.1	<0.001
Spine T-score	0.2 ± 0.9	−2.0 ± 0.7	<0.001
FN area	5.0 ± 0.5	5.0 ± 0.4	0.764
FN BMC (g)	3.8 ± 0.5	3.3 ± 0.4	<0.001
FN BMD (g/cm^2^)	0.76 ± 0.09	0.66 ± 0.07	<0.001
Hip area (cm^2^)	35.2 ± 3.8	34.8 ± 3.0	0.430
Hip BMC (g)	32.4 ± 5.2	27.6 ± 3.8	<0.001
Hip BMD (g/cm^2^)	0.92 ± 0.11	0.79 ± 0.77	<0.001
Hip T-score	−0.2 ± 0.9	−1.2 ± 0.7	<0.001
Whole body area (cm^2^)	2029.1 ± 134.2	1881.9 ± 112.0	<0.001
Whole body BMC (g)	2419.7 ± 318.7	2010.7 ± 201.4	<0.001
Whole body BMD (g/cm^2^)	1.19 ± 0.10	1.07 ± 0.08	<0.001
Whole body TFM (kg)	32.2 ± 8.2	26.7 ± 7.6	<0.001
Whole body TLM (kg)	42.6 ± 4.6	38.8 ± 3.6	<0.001
TFM/TLM ratio	0.8 ± 0.2	0.7 ± 0.2	0.031
Waist/hip ratio	0.8 ± 0.1	0.8 ± 0.1	0.009
Stiffness index	93.7 ± 13.7	84.5 ± 12.0	<0.001
QUS T-score	−0.4 ± 0.9	−1.0 ± 0.8	<0.001
QUS Z-score	1.0 ± 0.8	0.5 ± 0.8	<0.001
AEE (cal/min)	4724.0 ± 14380.7	1516.5 ± 5483.9	0.097

Similar to Table 2, a *t*-test was performed to observe the contribution of BMI to LM and FM. A total of 79 women with BMI ≥25 kg/m^2^ had significantly higher weight, BMI, WC, HC, BMC, BMD, and T-score but lower AEE than those with a BMI <25 kg/m^2^ (*n* = 46) as can be observed in Table 3. Women with BMI ≥25 kg/m^2^ (FM [M = 33.9, SD = 6.2] and LM [42.0, SD = 4.3]) had significantly higher body compositions than those with BMI <25 kg/m^2^ (FM [M = 21.5, SD = 4.9] and LM [M = 38.3, SD = 4.0]); FM, *t*(123) = −11.50, *p* < 0.001 and LM, *t*(123) = −4.72, *p* < 0.001 (Table 3).

**Table 3.** **Comparison of Anthropometric and Dual-Energy X-Ray Absorptiometry Data of Subjects with Body Mass Index (BMI) <25 kg/m^2^ and BMI ≥25 kg/m^2^**

	BMI <25 kg/m^2^ (*n* = 46)	BMI ≥25 kg/m^2^ (*n* = 79)	Independent *t*-test
Parameters	Mean ± SD		*p*
Age (years)	62.1 ± 4.2	62.9 ± 4.7	0.328
Weight (kg)	58.8 ± 6.5	75.4 ± 8.6	<0.001
Height (cm)	162.5 ± 5.4	162.2 ± 5.3	0.796
BMI (kg/m^2^)	22.3 ± 2.0	28.7 ± 3.2	<0.001
WC (cm)	71.2 ± 6.9	86.5 ± 8.4	<0.001
HC (cm)	92.9 ± 5.6	103.1 ± 6.0	<0.001
Spine area (cm^2^)	57.7 ± 5.6	57.2 ± 6.3	0.639
Spine BMD (g/cm^2^)	0.86 ± 0.13	0.99 ± 0.15	<0.001
Spine BMC (g)	50.0 ± 10.5	56.6 ± 11.3	0.002
Spine T-score	−1.7 ± 1.2	−0.5 ± 1.3	<0.001
FN area	5.0 ± 0.4	5.0 ± 0.5	0.648
FN BMC (g)	3.4 ± 0.5	3.7 ± 0.5	0.002
FN BMD (g/cm^2^)	0.67 ± 0.08	0.73 ± 0.96	0.001
Hip area (cm^2^)	34.6 ± 3.4	35.2 ± 3.4	0.312
Hip BMC (g)	27.7 ± 4.6	31.2 ± 5.0	<0.001
Hip BMD (g/cm^2^)	0.80 ± 0.10	0.88 ± 0.11	<0.001
Hip T-score	−1.1 ± 0.9	−0.5 ± 0.9	<0.001
Whole body area (cm^2^)	1885.0 ± 144.7	1991.9 ± 127.5	<0.001
Whole body BMC (g)	2081.1 ± 288.5	2280.4 ± 338.0	0.001
Whole body BMD (g/cm^2^)	1.10 ± 0.10	1.14 ± 0.11	0.052
Whole body TFM (kg)	21.5 ± 4.9	33.9 ± 6.2	<0.001
Whole body TLM (kg)	38.3 ± 4.0	42.0 ± 4.3	<0.001
TFM/TLM ratio	0.6 ± 0.1	0.8 ± 0.1	<0.001
Waist/hip ratio	0.8 ± 0.1	0.8 ± 0.1	<0.001
Stiffness index	87.0 ± 12.7	90.0 ± 14.0	0.235
QUS T-score	−0.8 ± 0.8	−0.6 ± 0.9	0.239
QUS Z-score	0.6 ± 0.8	0.9 ± 0.9	0.124
AEE (cal/min)	5187.8 ± 16,967.4	1814.9 ± 3858.7	0.092

In Table 4, there were positive correlations between the body composition variables and all the BMD measures at different sites as well as the QUS T-score. Negative correlations were observed with the women's age and all the BMD measurements and QUS T-score. In contrast, LM had higher significant positive correlations with BMD at all sites than FM. Likewise, high significant positive correlations were observed for weight and all BMD sites.

**Table 4.** **Pearson Correlation Coefficients of Body Composition Parameters and Bone Mineral Density**

Parameters	Femoral neck BMD (g/cm^2^)	Hip BMD (g/cm^2^)	Spine BMD (g/cm^2^)	Whole body BMD (g/cm^2^)	QUS T-score
Age (years)	−0.282^**^	−0.271^***^	−0.023^ns^	−0.151^*^	−0.262^**^
Weight (kg)	0.468^***^	0.537^***^	0.455^***^	0.305^***^	0.278^**^
Height (cm)	0.259^**^	0.196^*^	0.089^ns^	0.219^**^	0.240^**^
WC	0.276^**^	0.387^***^	0.405^***^	0.141^ns^	0.098^ns^
HC	0.286^**^	0.349^***^	0.299^***^	0.145^*^	0.136^ns^
Waist-hip ratio	0.157^*^	0.254^**^	0.312^***^	0.071^ns^	0.024^ns^
BMI (kg/m^2^)	0.367^***^	0.463^***^	0.427^***^	0.205^*^	0.193^*^
FM (g)	0.346^***^	0.446^***^	0.377^***^	0.191^*^	0.134^ns^
LM (g)	0.497^***^	0.495^***^	0.449^***^	0.406^***^	0.387^***^
Stiffness index	0.551^***^	0.520^***^	0.410^***^	0.437^***^	0.999^***^
QUS T-score	0.549^***^	0.516^***^	0.408^***^	0.433^***^	1.000
QUS Z-score	0.479^***^	0.451^***^	0.411^***^	0.408^***^	0.953^***^
AEE (cal/min)	0.103^ns^	0.053^ns^	0.185^*^	0.092^ns^	0.247^**^

^*^ *p* < 0.05; ^**^p < 0.01; ^***^p < 0.001 (one tailed).

FM, fat mass; LM, lean mass; ns, not significant.

Finally, to test if LM, FM, age, BMI, and AEE significantly predicted BMD at all sites in Table 5, multiple regression was used. The analysis shows that LM accounts for 24.7% of the variation in FN BMD (*F*[1, 123] = 40.3, *p* < 0.001). Furthermore, the introduction of age explains an additional 5.4% of the variation (*F*[2, 122] = 26.3, *p* < 0.001). For hip BMD, observations in Table 5 show that three predictors explained 35.5% of the variance (*F*[3, 121] = 22.2, *p* < 0.001). It was found that LM significantly predicted hip BMD (β = 0.348, *p* < 0.001) as did FM (β = 0.275, *p* < 0.01) and age (β = −0.219, *p* < 0.01). Similarly, three predictors explained 28.5% of the variation for the spine BMD (*F*[3, 121] = 16.0, *p* < 0.001). LM significantly predicted spine BMD (β = 0.243, *p* < 0.05) as well as BMI (β = 0.325, *p* < 0.01) and AEE (β = 0.196, *p* < 0.05). Conversely, only LM explained 16.5% of the variability in whole body BMD (*F*[1, 123] = 24.3, *p* < 0.001). The LM significantly predicted whole body BMD (β = 0.406, *p* < 0.001).

**Table 5.** **Multiple Regression Analysis Showing Predictors of Bone Mineral Density**

	B	SE B	95% CI B	β	*R*^2^	*p*
FN BMD						
Model 1					0.247	<0.001
Intercept	0.288	0.067	0.155 to 0.421			
LM	1.04 × 10^−5^	0.000	0.000 to 0.000	0.497		
Model 2					0.301	<0.001
Intercept	0.620	0.126	0.371 to 0.869			
LM	9.90 × 10^−6^	0.000	0.000 to 0.000	0.473		
Age	−0.005	0.002	−0.008 to −0.002	−0.234		
Hip BMD						
Model 1					0.245	<0.001
Intercept	0.350	0.080	0.192 to 0.509			
LM	1.24 × 10^−5^	0.000	0.000 to 0.000	0.495		
Model 2					0.307	<0.001
Intercept	0.367	0.077	0.214 to 0.520			
LM	9.22 × 10^−6^	0.000	0.000 to 0.000	0.369		
FM (g)	3.80 × 10^−6^	0.000	0.000 to 0.000	0.279		
Model 3					0.355	<0.001
Intercept	0.738	0.145	0.451 to 1.025			
LM	8.71 × 10^−6^	0.000	0.000 to 0.000	0.348		
FM	3.75 × 10^−6^	0.000	0.000 to 0.000	0.275		
Age	−0.006	0.002	−0.009 to −0.002	−0.219		
Spine BMD						
Model 1					0.202	<0.001
Intercept	0.332	0.110	0.115 to 0.550			
LM	1.50 × 10^−5^	0.000	0.000 to 0.000	0.449		
Model 2					0.250	<0.001
Intercept	0.274	0.109	0.058 to 0.490			
LM	1.03 × 10^−5^	0.000	0.000 to 0.000	0.309		
BMI	0.009	0.003	0.003 to 0.016	0.261		
Model 3					0.285	<0.001
LM	8.12 × 10^−6^	0.000	0.000 to 0.000	0.243		
BMI	0.012	0.003	0.005 to 0.019	0.325		
AEE	2.75 × 10^−6^	0.000	0.000 to 0.000	0.196		
Whole body BMD						
Model					0.165	<0.001
Intercept	0.728	0.081	0.567 to 0.889			
LM	9.80 × 10^−6^	0.000	0.000 to 0.000	0.406		

CI, confidence interval; SE, standard error of the coefficient.

## Discussion

The results from this study indicate that there is a strong positive correlation between weight, BMI, and regional (FN, hip, and spine BMD), whole body BMD as well as the QUS T-score. Similarly, AEE was positively correlated with QUS T-score but not with the regional and whole body BMDs. LM, not FM, was found to be the strongest predictor of BMD at the regional sites and whole body. The multiple regression analysis showed that LM had significant positive regression weights, indicating that individuals with higher LM will be expected to have higher BMDs at all regional sites and whole body even after controlling for other variables in the model.

The BMI of the participants in this study is comparable to that of “A Focus on Nutrition: Key Findings of the 2008/09 New Zealand Adult Nutrition Survey.”^21^ According to WHO, 29.6% of adult females in Western Pacific are overweight compared to 24.1% in Southeast Asia and 60.9% in the Americas.^22^ In comparison with corresponding statistics, 29% of women in India, 27% in Oceania, and 12.1–17.6% in Latin America are osteoporotic.^23^ Although the populations need to be considered, the trend shows people with lower BMI are more likely to have bone health issues.

In this study, obesity was positively associated with bone mass. Meanwhile, age was identified as a copredictor for FN and hip BMD, likewise BMI and AEE for spine BMD. Similarly, Salamat et al.^24^ found a positive correlation between BMD and BMI indicators, giving additional evidence for the obesity paradox. Some studies have reported that obesity is positively associated with high bone mass^25,26^ probably as a result of the increased levels of hormones such as leptin, insulin, and estrogen that are known to induce bone growth and inhibit the bone remodeling process. Other studies, however, have reported that obesity was negatively associated with bone mass,^5,27^ possibly due to the differences in patterns and occurrence of obesity, fat distribution, and osteoporosis in men and women, and between pre- and postmenopausal women.^6^

Furthermore, the results of this study show that LM alone accounts for 24.7% of FN, 24.5% of hip, and 20.2% of the spine BMDs' variability. These findings are similar to that of Casale et al.^13^ and Sotunde et al.^12^ in Pacific Island and black South African women, respectively, indicating that LM is the strongest predictor of BMD. Our result also suggests that LM is more positively correlated with bone mass than the adipose tissue. A study by Povoroznyuk et al.^28^ in Ukraine presented a similar result showing a positive correlation between the total lean mass (TLM) and FN and spine BMD for women in the middle and late postmenopausal period. In addition, a study of postmenopausal women by Gnudi et al.^7^ shows that TLM and total fat mass were associated with BMD, BMC, and height-independent BMD in postmenopausal women. Similar results were also observed in a study by Wang et al.^29^; they found LM had a greater effect on BMD than FM in young women.

However, results of this study are contrary to previous studies by Reid et al.,^30–32^ suggesting that the relationship between LM and BMD are artifacts. The differences in these results, however, could be explained by the meta-analysis of Khosla et al.,^8^ which found that both lean body mass and fat body mass have important effects on bone mass, depending on the bone mass parameter used, the skeletal site measured, and menopausal status.

Limitations of this study include its cross-sectional design and setting, thus preventing causal relationships and generalization. The method of assessing physical activity was NZPAQ; however, bone-specific physical activity questionnaire has current and past bone-related exercises. Furthermore, there was lack of other contributors and predictors of bone status such as diet, nutrients, and vitamin D.

## Conclusion

In conclusion, our findings suggest that LM is the strongest predictor of BMD at all sites. It is important that when considering prevention and/or management of osteoporosis, LM should be the target for improvement rather than FM reduction. In addition, it emphasizes the significance of the accumulation of LM rather than FM in this age group. These findings will bring about further novel clinical research on the mechanisms by which LM regulates bone mass.

## Abbreviations Used

AEE: activity energy expenditure
BMC: bone mineral content
BMD: bone mineral density
BMI: body mass index
CI: confidence interval
DXA: dual-energy X-ray absorptiometry
FM: fat mass
FN: femoral neck
HC: hip circumference
LM: lean mass
LS: lumbar spine
ns: not significant
NZPAQ: New Zealand Physical Activity Questionnaire
QUS: quantitative heel ultrasound
SD: standard deviation
SE: standard error of the coefficient
TFM: total fat mass
TLM: total lean mass
WC: waist circumference
WHO: World Health Organization

## References

[1] Sànchez-Riera L, Carnahan E, Vos T, et al. The global burden attributable to low bone mineral density. Ann Rheum Dis. 2014;73:1635–1645 24692584 10.1136/annrheumdis-2013-204320

[2] World Health Organization. WHO Scientific Group on the Assessment of Osteoporosis at Primary Health Care Level. 2011. World Health Organization: Geneva, Switzerland, 2013

[3] Siris ES, Adler R, Bilezikian J, et al. The clinical diagnosis of osteoporosis: a position statement from the National Bone Health Alliance Working Group. Osteoporos Int. 2014;25:1439–1443 24577348 10.1007/s00198-014-2655-z PMC3988515

[4] Shils ME, Shike M Modern Nutrition in Health and Disease. LippincottWilliams & Wilkins: Philadelphia, PA, 2006

[5] Hsu YH, Venners SA, Terwedow HA, et al. Relation of body composition, fat mass, and serum lipids to osteoporotic fractures and bone mineral density in Chinese men and women. Am J Clin Nutr. 2006;83:146–154 16400063 10.1093/ajcn/83.1.146

[6] Ho-Pham LT, Nguyen UD, Nguyen TV Association between lean mass, fat mass, and bone mineral density: a meta-analysis. J Clin Endocrinol Metab. 2014;99:30–38 24384013 10.1210/jc.2014-v99i12-30A

[7] Gnudi S, Sitta E, Fiumi N Relationship between body composition and bone mineral density in women with and without osteoporosis: relative contribution of lean and fat mass. J Bone Miner Metab. 2007;25:326–332 17704998 10.1007/s00774-007-0758-8

[8] Khosla S, Atkinson EJ, Riggs BL, et al. Relationship between body composition and bone mass in women. J Bone Miner Res. 1996;11:857–863 8725184 10.1002/jbmr.5650110618

[9] Durnin JV, Womersley J Body fat assessed from total body density and its estimation from skinfold thickness: measurements on 481 men and women aged from 16 to 72 years. Br J Nutr. 1974;32:77–97 4843734 10.1079/bjn19740060

[10] Follin SL, Hansen LB Current approaches to the prevention and treatment of postmenopausal osteoporosis. Am J Health Syst Pharm. 2003;60:883–901; quiz 903 12756940

[11] Zhao LJ, Liu YJ, Liu PY, et al. Relationship of obesity with osteoporosis. J Clin Endocrinol Metab. 2007;92:1640–1646 17299077 10.1210/jc.2006-0572 PMC1868430

[12] Sotunde OF, Kruger HS, Wright HH, et al. Lean mass appears to be more strongly associated with bone health than fat mass in urban black South African women. J Nutr Health Aging. 2015;19:628–636 26054499 10.1007/s12603-015-0492-1 PMC12877595

[13] Casale M, von Hurst PR, Beck KL, et al. Lean mass and body fat percentage are contradictory predictors of bone mineral density in pre-menopausal Pacific Island women. Nutrients. 2016;8:470 10.3390/nu8080470 PMC4997383 27483314

[14] Liu J-M, Zhao H-Y, Ning G, et al. Relationship between body composition and bone mineral density in healthy young and premenopausal Chinese women. Osteoporos Int. 2004;15:238–242 14727013 10.1007/s00198-003-1536-7

[15] Rodrigues Filho EdA, Santos MAMd, Silva ATPd, et al. Relation between body composition and bone mineral density in young undergraduate students with different nutritional status. Einstein (São Paulo). 2016;14:12–17 27074228 10.1590/S1679-45082016AO3569 PMC4872911

[16] Harris SS, Dawson-Hughes B Weight, body composition, and bone density in postmenopausal women. Calcif Tissue Int. 1996;59:428–432 8939766 10.1007/BF00369205

[17] Mizuma N, Mizuma M, Yoshinaga M, et al. Difference in the relative contribution of lean and fat mass components to bone mineral density with generation. J Obstet Gynaecol Res. 2006;32:184–189 16594922 10.1111/j.1447-0756.2006.00384.x

[18] Ijuin M, Douchi T, Matsuo T, et al. Difference in the effects of body composition on bone mineral density between pre- and postmenopausal women. Maturitas. 2002;43:239–244 12468131 10.1016/s0378-5122(02)00273-6

[19] Kim JH, Choi HJ, Kim MJ, et al. Fat mass is negatively associated with bone mineral content in Koreans. Osteoporos Int. 2012;23:2009–2016 22006041 10.1007/s00198-011-1808-6

[20] McLean G, Tobias M The New Zealand Physical Activity Questionnaires: Report on the Validation and Use of the NZPAQ-LF and NZPAQ-SF Self-Report Physical Activity Survey Instruments. SPARC: Wellington, New Zealand, 2004

[21] Parnell W, Wilson N, Thomson C, et al. A Focus on Nutrition: Key Findings of the 2008/09 New Zealand Adult Nutrition Survey. Ministry of Health: Wellington, New Zealand, 2011

[22] World Health Organization. Prevalence of overweight among adults, BMI ≥25, age-standardized: Estimates by WHO Region. 9 27, 2017 Available at: http://apps.who.int/gho/data/view.main.GLOBAL2461A?lang=en Accessed 615, 2018

[23] International Osteoporosis Foundation. Osteoporosis—Incidence and burden. 2017 Available at: https://www.iofbonehealth.org/facts-statistics Accessed 620, 2018

[24] Salamat MR, Salamat AH, Janghorbani M Association between obesity and bone mineral density by gender and menopausal status. Endocrinol Metab. 2016;31:547–558 10.3803/EnM.2016.31.4.547 PMC5195832 27834082

[25] Kim K-C, Shin D-H, Lee S-Y, et al. Relation between obesity and bone mineral density and vertebral fractures in Korean postmenopausal women. Yonsei Med J. 2010;51:857–863 20879051 10.3349/ymj.2010.51.6.857 PMC2995981

[26] Gower BA, Casazza K Divergent effects of obesity on bone health. J Clin Densitom. 2013;16:450–454 24063845 10.1016/j.jocd.2013.08.010 PMC5321047

[27] Zhao LJ, Jiang H, Papasian CJ, et al. Correlation of obesity and osteoporosis: effect of fat mass on the determination of osteoporosis. J Bone Miner Res. 2008;23:17–29 17784844 10.1359/JBMR.070813 PMC2663586

[28] Povoroznyuk V, Ivanyk O, Dzerovych N Bone mineral density and quality, body composition of women in the postmenopausal period. Maturitas. 2017;100:157

[29] Wang MC, Bachrach LK, Van Loan M, et al. The relative contributions of lean tissue mass and fat mass to bone density in young women. Bone. 2005;37:474–481 16040285 10.1016/j.bone.2005.04.038

[30] Reid IR, Evans MC, Ames RW Volumetric bone density of the lumbar spine is related to fat mass but not lean mass in normal postmenopausal women. Osteoporos Int. 1994;4:362–367 7696834 10.1007/BF01622199

[31] Reid IR, Ames R, Evans MC, et al. Determinants of total body and regional bone mineral density in normal postmenopausal women—a key role for fat mass. j Clin Endocrinol Metab. 1992;75:45–51 1619030 10.1210/jcem.75.1.1619030

[32] Reid IR, Plank LD, Evans MC Fat mass is an important determinant of whole body bone density in premenopausal women but not in men. J Clin Endocrinol Metab. 1992;75:779–782 1517366 10.1210/jcem.75.3.1517366

